# Sudden Death of Isaac F. Bryant from Supposed Heart Disease—Autopsy—Comments

**Published:** 1869-12

**Authors:** C. C. F. Gay


ART. III.—*Sudden death of* ISAAC F. BRYANT *from supposed Heart Disease—Autopsy—Comments.* BY C. C. F. GAY, M. D.

On the morning of Monday, the 15th of November, 1869, Mr. Isaac F. Bryant, a most estimable citizen, fell upon the sidewalk on Exchange Street, opposite the Central Rail Road passenger depot, was immediately taken up in an attitude midway between the upright and recumbent position, and carried across the street, and laid upon a sofa in the National Hotel, where, after two or three gasps, he expired.

A coroner and two physicians were summoned, whose verdict, after examination, informally rendered, was "death from heart-disease."

Mr. Bryant was forty-nine years of age, of about two hundred pounds weight, of temperate habits, and had always enjoyed the best of health. On the morning of his sudden death he arose in good health and spirits, ate a hearty breakfast, and rode into the city from Lancaster, a distance of ten miles, conversing cheerfully with friends on the way. He alighted from the cars at the depot of the Erie Rail Road and walked, in company with a stranger, until he arrived opposite the passenger depot of the Central Rail Road, when he turned suddenly around, with his eyes rolled upwards, and fell at full length, striking and inflicting a severe wound upon the forehead about one inch above the right eye. Mr. Bryant, a few days prior to his death, had been examined for life insurance by a competent physician, who tells me that, at the time of examination, the pulse was seventy-four per minute, and that he recommended him to the company as a safe risk. I may statc, in this place, that the policy for three thousand dollars was not delivered until after death, and that the Washington Life Ins. Co., of New York, on learning the death of Mr. Bryant, very creditably to itself, immediately caused the policy to be paid through the agents, the Messrs. Parsons of this city.

By request of the brother of deceased, I held an autopsy, twenty-four hours after post-mortem, assisted by Drs. Whitney, Boardman, Hopkins and Chase.

Rigor mortis well marked, hypostatic congestion extending from neck along down the spinal column, neck very flexible, suggestive of fracture.

The undertaker had observed that semen had been discharged, which fact pointed toward probability of fracture at the neck. Accordingly we cut down upon the cervical vertebræ and removed the axis, together with the third cervical vertebra. Although there were evidences of some injury here, manifested by the congestion at upper portion of the spinal column, there were no evidence of fracture of any one of the cervical vertebræ.

In the next place the viscera of the thorax were exposed, the pericardium opened, and found to contain no effusion. The heart was removed and carefully examined; it was scarcely, if any, enlarged; it was slightly softened; there was no valvular lesion. The heart was filled with fluid blood; there was no coagulum nor heart clot. The other viscera of the body were normal.

We next removed the calvaria, and found the brain and its inverting membranes in a normal condition.

COMMENTS.—The examination disproved the theory that the cause of death was heart disease. The most that can be said to sustain the theory that death began at the heart, is the fact that the heart was full of fluid blood. Sometimes, when the heart is thus filled, the organ is unable to contract down upon its fluid contents, and death immediately ensues; but the post-mortem examination showed no such inability in the power of the heart. The softening observable was no greater in degree than could be expected to exist forty-eight hours after death. The partial enlargement was not from dilatation; and the heart possessed, undoubtedly, the power of any other heart in a normal condition to contract down upon and expel its fluid contents, hence the verdict informally rendered, without a post-mortem examination, was not sustained by the post-mortem examination.

The emission of semen, observable in those who are hung, led some friends of deceased to think that, perhaps, the neck had been broken in the fall; and, as evidence of such theory, cited the fact of the emission of the seminal fluid. Although the neck was very flexible and much discolored, the post-mortem examination furnished no positive evidence either of dislocation, fracture, or of sufficient injury to cause immediate death.

A fracture of any one of the cervical vertebræ is one of the rarest accidents known, nevertheless fracture of these vertebræ do occur; but, whenever such fractures occur, they very rarely cause sudden death.

Dr. Frank H. Hamilton, in his valuable work on "Fractures and Dislocations," page 151, says, that "in nine recorded examples of fractures of the five lower cervical vertebræ, one died in twenty-four hours; but that the most common period of death seems to be about forty-eight hours after the receipt of injury."

On page 155, Dr. Hamilton says, that "the phrenic nerve is derived chiefly from the third and fourth cervical nerves. If, therefore, the second cervical vertebra is broken, and considerably depressed upon the spinal cord, respiration ceases immediately, and the person dies at once, or survives only a few minutes."

A case is recorded by Dr. H. of "a woman who, while sitting in bed eating, was seen to fall suddenly forward, and the patients of St. Thomas Hospital, of which she was an inmate, hastened to her and found her dead. Upon examination of her body, it was discovered that the processus dentatus of the axis was broken off, and that the head, in falling forwards, had driven the process backwards upon the spinal marrow, so as to cause her death."

We removed from the neck, at this post-mortem examination, the axis; and I have it in my possession. There is no fracture of the odontoid process, nor of any other portion of the bone.

Dr. H. says: "I have been able to find only one example of a fracture of the atlas bone; and this is the case related by Sir. Astley Cooper. It occurred in a boy three years of age, and who lived one year after the injury."

I have made these quotations from the best standard work upon the subject of which it treats, to show the variety of accidents which result in fracture of the bones of the neck; and to show, also, in the same connection, that such accidents do not usually result in sudden death.

Discovering no cause of death either in this locality nor in the heart, I surely thought of being successful on examination of the brain. I was more impressed with the belief that cerebral effusion, or a blood clot, would be revealed, on exposing the brain, from the coincidence of having seen, just about the time of the death of Mr. Bryant, three persons, viz., Dr. Bristol, Mrs. D. Lockwood, and Mrs. Levi, aged, respeetively, 77, 55, and 32 years, who were suddenly stricken down with paralysis, the result of brain affection, and all three of whom died within a few hours from their attack, remaining unconscious and insensible from the first moment of attack. The physical conformation of the person whose death is the subject of these comments was such as would predispose to apoplexy; yet, if his attack had been of this character, immediate death would not have been looked for. The post-mortem examination cleared up all doubts and conjectures as to cause of death being from apoplectic seizure.

The brain and the heart, with their investing membranes, and the bones of the neck, having been found in their integrity, each and all being excluded as cause of sudden death, the question then recurs as to the true and undoubted cause.

This question I am to try to answer; and the answer shall not be altogether conjectural, I trust, since science is enabled to furnish sufficient data by which very accurately to arrive at the explanation of the theory of the cause of death and where death began.

Stated in brief, the cause of Mr. Bryant's death was from severe concussion immediately following faintness or syncope. Either the one or the other alone would be sufficient to cause death; but, the two conjoined, would be most surely fatal, provided certain contingencies existed, of which mention will be made further on. Concussion, in this case, was produced by a blow upon the forehead from a fall. The cause of syncope is conjectural only. Very slight causes often produce faintness. I have known the most robust persons to faint from vaccination, when not a drop of blood had been drawn. Sudden severe pain will cause it. I may suppose, in the case under review, that Bryant may have made a mistep, wrenching his foot or ankle joint in such a manner as to cause a sudden twing of pain. I know of persons of sound health and hearts who faint on the slightest provocation, without any apparent or efficient cause; indeed, so many cases of syncope almost daily witnessed, and so many cases of deaths from supposed heart disease, are reported through the medium of the daily papers, that the question of the true cause of Bryant's death becomes a question of public interest. Cases are well authenticated of sudden death from a blow upon the pit of the stomach; add to the concussion by a blow at the stomach, or upon the head, syncope, and the person is in imminent peril of his life, and would most likely die suddenly, provided prompt measures were not had recourse to for his resuscitation. It is the experience of many persons—indeed almost every person has, during the course of his life time, had his breath "knocked out" of his body by a blow, either upon his head or over the stomach; and such persons know full well the distress which attends such concussion, and the difficulty he labors under, momentarily, in regaining the respiratory function.

Assuming, in any given case, the presence of the following two conditions, would the suspension of the circulation, and of respiration, simultaneously, preclude the possibility of restoration of these two functions again to their normal condition?

I answer that resuscitation is not at all incompatible with such conditions. It must, unfortunately, be confessed, however, that popular intelligence is wanting in respect to the care and management of such as fall from faintness.

There are published rules to be made available by any one for resuscitation from drowning and for the temporal treatment and management of the injured by rail road accident; and it were well if published rules were placed in the hands of all persons to be made available in aiding and assisting those who fall from faintness or concussion.

The natural remedy for syncope is the horizontal position. If unmolested and unobstructed, the body at once falls into this attitude, the heart is thereby assisted in carrying along its current of blood, and, if let alone, the person immediately revives.

It is mistaken kindness, and the result of ignorance, to sieze a person who may have fallen from syncope and place him in the upright position. Kind hearted people, and those who are not kind hearted, will forever do this thing because they do not know the danger of it; and certainly no blame attaches such misapplied acts of kindness.

In the case of Mr. Bryant, he was taken up, carried across the street in a semi-recumbent position, laid upon the sofa, after which he gave two or three gasps and expired. These friends performed their duty as they understood it, and there is no one who can have the hardihood to censure them for the act. Had they not have done it there were others in readiness to do it; and, however trying and indelicate for one to make criticism upon acts in themselves humane, the startling fact, nevertheless, remains, and must not be lost sight of, because of prospective benefits to accrue to others, that such acts of humanity deprive the person of the sole and only means which nature furnishes for relief from syncope.

It will always be a good rule to follow when one has fallen from faintness, in absence of a physician, to let the person lie in the horizontal position; and, if respiration be also suspended, to turn him, as all physicians well understand, gently and alternately upon his side and back from twelve to sixteen times a minute, corresponding to the number of inspirations per minute, which constitutes the "Marshall Hall method" cf re-establishing respiration after its temporary suspension. Post-mortem examination furnishing no evidence to the contrary, it is not unreasonable to conclude that, had the rules herein stated been observed, they would have been crowned with success in the restoration of Bryant to health and life.

